# Analysis and determinants of Chinese navy personnel health status: a cross-sectional study

**DOI:** 10.1186/s12955-018-0961-4

**Published:** 2018-07-11

**Authors:** Shali Xie, Hui Lin, Yi Meng, Jundong Zhu, Yanqi Zhang, Ling Zhang, Gaoming Li

**Affiliations:** 1 Department of Health Education, College of Military Preventive Medicine, Army Medical University, Chongqing, 400038 China; 2 Department of Tropical Epidemiology, College of Military Preventive Medicine, Army Medical University, Chongqing, 400038 China; 3 Department of Social Work, Social and Public Management School, Chongqing Technology and Business University, Chongqing, 400067 China; 4 Department of Nutrition and Food Hygiene, College of Military Preventive Medicine, Army Medical University, Chongqing, 400038 China; 5 Department of Health Statistics, College of Military Preventive Medicine, Army Medical University, Chongqing, 400038 China

**Keywords:** Health status, Health related behaviors, Navy, Veterans SF-36, Military, China

## Abstract

**Background:**

There have been very few studies analyzing the relationship of physical and mental health status with health behaviors and deployment status in Chinese navy personnel. Thus, we undertook this survey to assess this relationship and identify specific factors affecting the physical and mental health status.

**Methods:**

The subjects enrolled in this study were selected from four units of the active-duty navy personnel in China, based on a cluster random sampling design. A total of 1200 Chinese navy personnel participated and completed the questionnaire survey that included veteran SF-36 form and a self-designed questionnaire regarding their sociodemographic characteristics, deployment status, self-rated health status and health behaviors. Totally 1200 questionnaires were distributed to different participants, while 1083 valid questionnaires were included in the final analysis. All data were analyzed using SPSS18.0 software.

**Results:**

Based on the information provided by navy personnel, 17.82, 35.09 and 23.08% rated their health as excellent, very good and good, respectively. The mean score of physical component summary (PCS) and mental component summary (MCS) was 50.53 and 41.39, respectively. Length of service, binge drinking, regular drinking and BMI appeared to be associated with PCS score, while household income, binge drinking and BMI affected MCS score. Deployment status and smoking exhibited no significant association with PCS and MCS scores.

**Conclusions:**

Our study suggested that the sociodemographic factors like length of service and household income, along with behavioral risk factors like binge drinking, regular drinking and body mass index (BMI), seem to affect the physical and mental health status of Chinese navy personnel. However, additional data collection and more detailed analysis would still be required to develop a systematic, comprehensive and corresponding health education program to promote overall health status.

## Background

Military operations and related tasks require military personnel to maintain optimal physical and mental health fitness. Despite significant progress in defense technology, there remains a demand for high levels of physical fitness and physical functioning capacity [1]. Despite the younger average age of Chinese navy personnel in comparison with general population, there is still a need to explore their health behaviors and modifiable risks, and to understand levels of physical functioning and factors associated with higher levels of fitness [2].

Many behavioral factors or health habits, such as cigarette smoking, alcohol consumption, physical exercise, weight gain, eating habits and hours of sleep, were significantly associated with physical well-being and overall health. Among these factors, smoking and drinking are more prominent factors in the military population reported by many studies [2, 3]. For example, Kaly et al. [3] and LaBrie et al. [4] reported that active duty military personnel drink more heavily than their civilian counterparts and also engage in other health risk behaviors such as smoking, substance abuse and risky sexual behaviors. A survey on the alcohol drinking habit of Chinese naval pilots by Zhang et al. [5] reported that 74% of the survey population was drinking, and most of them started using alcohol after they joined the army, thereby implying that this can obviously affect flight safety and increase the risk of accidents. Specific smoking has been reported to be a major issue due to its high rate, which eventually impairs the troop readiness and productivity and increases the medical and training costs [6, 7]. An investigation on smoking behavior of Chinese naval aviators showed that total smoking rate was about 61%, and of which 42% smokers suffered from one or more smoking-related diseases [8]. However, above two studies only focused on naval aviators in China, and little information is known about the prevalence of smoking and drinking in all branches of Navy. Moreover, other health risks including weight gain in military personnel as reported by studies in other countries, primarily due to poor dietary patterns and sedentary lifestyles in some cases, have been strongly linked with increased prevalence of acute and chronic illness, injury, healthcare costs, disability and absenteeism [9–11]. One domestic study described overweight or obesity along with other factors to be associated with obesity-related disorders such as hyperlipidemia and hyperuricemia in Chinese naval aviators [12]. However, no report was found regarding the association between weight gain and health status in Chinese naval personnel.

Moreover, the contextual factors affecting physical and mental health among general as well as military population have been well studied. Previous studies have indicated that demographic factors such as social support, marital status, household income and occupation significantly affect health outcomes [13]. For example, social support from financial assistance (e.g., household income), companionship and so on, shows a positive relationship with good perception of physical health and improving subjective well-being [13]. In addition, Smith et al. [1] found that being married and having a higher military rank are independently associated with more favorable health status. Other studies have indicated that military deployment is associated with adverse health related outcomes, including high-risk behavior and psychological morbidity from both real and perceived exposure [14, 15]. But, there has been little information about the exact relationship between the deployment status and self-rated health in the Chinese navy personnel.

In summary, it is important to understand the various demographic and behavioral factors adversely affecting health. This would subsequently lead to the development of comprehensive and cost-effective intervention programs for health promotion. Therefore, based on previous studies, we proposed the following hypotheses: (1) The health status of navy personnel is different from that of the general population, (2) certain sociodemographic factors and health hazard behaviors could influence their health status, and (3) military deployment is related to their health outcomes. Thus, in this study we investigated the health status of Chinese navy personnel, and explored the relationship between health status and sociodemographic factors, health-related behaviors and deployment status in a military cohort. We believe that these findings would be helpful in understanding the health of Chinese navy personnel, and can be useful in multiple ways like: (1) they can provide an indication about the quality of life of Chinese navy personnel, (2) specific information of health influencing factors can provide reference for their health education and health interventions, (3) these population-based results can also be used to compare the health of personnel within the navy or army or air force or with Chinese general population, (4) these results can also serve as a reference for exploring the health of particular sub groups within the military, such as the marines or coast guard, (5) the health status information can also provide a useful and comprehensive assessment of the health characteristics of these navy personnel and will be helpful in improving the health care delivery systems, along with their utility in the development of health promotion efforts.

## Methods

### Participants

This study was a cross-sectional survey of Chinese Navy personnel conducted between July and August 2016. The participants were recruited from four units of the active-duty navy members by cluster random sampling. Subjects who were not around when we conducted the survey were excluded from this study (*n* = 2, due to illness and unable to reach the site). Our investigators explained to the participants the purpose of the survey, and how to fill out the questionnaire and the specific points for attention. Next, the participants were asked to fill out and return the questionnaire, and a total of 1200 participants participated in this exercise with all of them providing written informed consent. All the responses were anonymous and voluntary. The participation rate was 99.83%. The study was conducted in compliance with the Helsinki Declaration and approved by the Ethics Committee of Army Medical University.

### Measurement

#### Veterans SF-36 form based data collection

Participants’ health status and health-related quality of life (HRQOL) were evaluated using the Short-Form 36 for veterans (SF-36 V), a version of the Short Form 36 (SF-36) health survey questionnaire, modified for use in military personnel [1, 16–18]. The SF-36 measures eight health areas: physical functioning (PF), role limitation due to physical problems (RP), body pain (BP), general health (GH), role limitation due to emotional problems (RE), mental health (MH), energy/vitality (VT), and social functioning (SF). Each area was scored from 0 to 100. SF-36 V differs from the original SF-36 as follows: RP and RE subscales are changed from a dichotomous scale to a 5-point scale to avoid floor and ceiling effects [19, 20] and improve the reliability and validity of the original version [18, 21]. Physical Component Summary (PCS) and Mental Component Summary (MCS) scores, which represent subjective physical health and mental health respectively, were calculated from eight subscale scores by using standard SF-36 scoring mechanism as recommended [19]. PCS and MCS were norm-based and population-standardized with 0–100 scoring range, mean of 50, and standard deviation of 10. Higher scores on subscale and summary scales indicate better functioning in that domain and better health status or HRQOL [1]. SF-36 V was found to have high internal consistency across all eight domains in military populations [18, 21]. The self-rated health was assessed based on the response to the following question, “In general, would you say your health is excellent, very good, good, fair or poor?” The answers to this question were classified into two categories: a) excellent/very good/good, and b) fair/poor.

### Assessment of sociodemographic, body mass index (BMI) and behavioral risk factors

To measure the health determinants, we used self-designed questionnaires to investigate demographic information, BMI and behavioral risk factors. The sociodemographic attributes included age, gender, nationality, marital status, education level, household income, residential location, and military-related information such as length of service, rank, and deployment status. Length of service was divided into four grades: 0-3 years, 4–8 years, 9–15 years, and > 16 years, according to the period of rank. The service for 9 years or above was defined as longer service time. BMI (kg/m^2^) was calculated from self-reported height and weight, was defined as underweight (<18.5 kg/m^2^), normal (18.5–23.9 kg/m^2^), overweight (24–27.9 kg/m^2^) and obese (≥28 kg/m^2^), according to Chinese population standards [22]. The behavioral variables included smoking and drinking. Smoking was grouped as smoked<20cigarettes/day, smoked≥20cigarettes/day, never smoked and past smokers. Heavy smokers referred to the smokers who smoked ≥20 cigarettes/day [23]. Drinking and binge drinking were grouped as everyday or almost every day, frequent, occasional and never. The frequencies were coded as follows: everyday or almost everyday = 6–7 times/week; Frequent = 1–5 times/week; Occasional = 3 times or below/month. Binge drinking represented five or more drinks in one sitting [24]. The deployment status, included war-related military operations and non-war military operation, was classified as without (i.e., the participants have no deployment experience prior to the study) or with (i.e., the participants have deployment experience prior to the study) experience.

### Data analysis

All data was entered in duplicate into the EpiData version3.1 database, and the SPSS18.0 software was used for data analysis. The data measurement was presented as mean ± standard deviation. The independent variables were expressed as a rate or constituent ratio using descriptive statistics. One-way ANOVA test was used to analyze these variables and determine the difference among multiple groups. Significant variables determined by univariate analysis were entered into final multivariable linear regression and logistic regression model to predict health status. A *p* value of< 0.05 was considered to be statistically significant.

## Results

### Demographic characteristics of the participants

The final participation rate was 99.83% (1200 of 1202). A total of 1200 questionnaires were distributed to different participants and they all submitted their answers, thereby indicating a response rate of 100%. Among the 1200 returned questionnaires, 117 questionnaires were excluded because of invalid or incomplete responses, and thus 1083 valid questionnaires were included in the analysis, indicating an effective rate of return as 90.25% (1083 of 1200). In 117 excluded questionnaires, except for one from a female soldier, the rest of 116 questionnaires were from males, aged 18–53 years, with an average age of 25.45 ± 8.79 years, and as to the rank, 33.33% of them were soldiers, 51.52% were sergeants, and 15.15% were officers. Their characteristics were not significantly different from those included in the analysis. Table 1 summarizes the demographic characteristics of the study population. Out of the 1083 participants, only 3 were females and the rest were males. The majority of the participants were Han population, less than 35 years old, with high school diploma or below, unmarried, from the countryside, and in service less than 9 years (78.30%). Most of them hold sergeants ranks (56.14%), followed by soldiers (30.93%), and officers (12.93%), which is consistent with the rank distribution in Chinese Navy. Therefore, the study is representing the whole Chinese Navy. Most of the participants had a household income of <4000 yuan/month (around 605 US dollar per month).

**Table 1:** Social demographic data (Number of respondents = 1083)

Characteristics	Number	Percent
Age(years)		
17~ 24	642	59.28
25~ 34	402	37.12
≥ 35	39	3.6
Race		
Han	1022	94.37
Minority	61	5.63
Length of service(years)		
0~ 3	473	43.67
4~ 8	375	34.63
9~ 15	197	18.19
≥ 16	38	3.51
Marital status		
Unmarried	847	78.21
Married, living together	95	8.77
Married, but separated	141	13.02
Education		
High school diploma or below	599	55.31
Some college	288	26.59
Bachelor’s degree or above	196	18.1
Residential Location		
Rural area	682	62.97
City	401	37.03
Rank		
Soldier	335	30.93
Sergeant	608	56.14
Officer	140	12.93
Household income(yuan/month)		
< 2000(~ 302 US dollar)	473	43.67
2000 to 4000(~ 302 to 605 US dollar)	439	40.54
> 4000(~ 605 US dollar)	171	15.79

### Self-reported health behaviors, military exposures and BMI of participants

As shown in Table 2, only less than 22% of the participants reported deployment experience, while most of participants reported no deployment experience. In addition, among the participants with modifiable risk behaviors, more than half of them were smokers, but most of them smoked<20 cigarettes/day. In the case of alcohol use, most of the participants reported drinking (82.09%), and nearly half of them (46.90%) reported to have been involved in binge drinking (five or more drinks in one sitting). Based on BMI, the majority of participants were normal (65.93%, *n* = 714).

**Table 2:** Self-reported military exposures and health-related indicators of participants

Variable	Number	Percent
Military exposures		
Past deployment status		
Deployment experience	233	21.51
No deployment experience	850	78.49
BMI (kg/m^2^)		
< 18.5	34	3.14
18.5~ 23.9	714	65.93
24.0~ 27.9	288	26.59
≥ 28.0	47	4.34
Health-related behaviors		
Smoking		
Smoked<20cigarettes/day	501	46.26
Smoked≥20cigarettes/day	63	5.82
Never smoked	463	42.75
Past smokers	56	5.17
Drinking		
Everyday or almost everyday	52	4.80
Frequent	209	19.30
Occasional	628	57.99
Never	194	17.91
Binge drinking		
Everyday or almost everyday	18	1.66
Frequent	108	9.97
Occasional	382	35.27
Never	575	53.10

Note: everyday or almost everyday = 6–7 times/week; Frequent = 1–5 times/week; Occasional = 3 times or below/month

### Self-rated health status along with PCS and MCS scores of participants

More than 50% of the participants reported very good or excellent physical health, as shown in Table 3. As for the Veterans SF-36 based assessment of functional status, mean scores of the 8 Veterans SF-36 scales ranged from 63.88 to 85.94, with the highest mean for physical functioning. Accordingly, the summary score of PCS was above the mean of 50 for the Chinese general population. However, MCS was about 8 point lower than Chinese general population, indicating a less favorable mental health state.

**Table 3:** Self-rated health status and PCS, MCS scores

Variable	Number	Percent
General health		
In general, would you say your health is		
Excellent	193	17.82
Very good	380	35.09
Good	250	23.08
Fair	235	21.7
Poor	25	2.31
SF-36 V	Mean ± SD	< Mean (%)
Physical		
PF	85.94 ± 19.88	346 (31.95)
RP	70.78 ± 26.50	475 (43.86)
BP	79.22 ± 21.02	521 (48.11)
GH	69.57 ± 19.91	509 (47.00)
Mental		
RE	72.31 ± 26.71	436 (40.26)
MH	64.50 ± 16.53	551 (50.88)
VT	63.88 ± 17.66	484 (44.69)
SF	77.11 ± 15.79	411 (37.95)
Summary scores^a^		
PCS	50.53 ± 9.24	448 (41.37)
MCS	41.39 ± 11.30	503 (46.46)

Note: ^a^standardized to Chinese population with a mean of 50 and standard deviation of 10; higher scores denote better health

### Effect of social demographic factors on the health status of study participants

To assess the effect of sociodemographic factors on the health status of the participants, we performed a univariate analysis, as shown in Table 4. Except race, age, marital status, educational level, and residential location, the factors that showed significant (*P* < 0.01) association with PCS score were the length of service and rank, while the rank and household income showed significant association with MCS score (*P* < 0.05). The participants who were in a longer service time had lower PCS score. Especially the PCS score in the participants over 9 years of service was significantly lower than those with fewer than 3 years of service (*P* < 0.05). Interestingly, the PCS score in the sergeants was significantly lower than that in the soldiers and officers (*P* < 0.05). As to MCS, the participants with higher household income displayed higher MCS score, especially group with >4000 yuan income had significantly higher MCS score than the group with < 2000 yuan (*P* < 0.05). The sergeant’s MCS score was lowest, and significantly lower than the officers (*P* < 0.05).

**Table 4:** Univariate analysis of sociodemographic items related to PCS and MCS scores among the participants

Variable	PCS			MCS		
	Mean ± SD	Levene	F/*χ*^2^(Z)	Mean ± SD	Levene	F/*χ*^2^(Z)
Race		0.28	F = 0.66		0.01	F = 0.86
Han	50.58 ± 9.19			41.31 ± 11.29		
Minority	49.58 ± 10.11			42.71 ± 11.44		
Age(years)		0.71	F = 2.24		0.01	F = 0.64
17~ 24	50.83 ± 9.47			41.03 ± 11.19		
25~ 34	50.21 ± 8.96			41.83 ± 11.52		
≥ 35	47.54 ± 8.10			41.82 ± 11.08		
Length of service(years)		0.40	F = 3.848**		0.49	F = 0.59
0~ 3	51.26 ± 9.56			41.12 ± 11.15		
4~ 8	50.44 ± 8.88			41.97 ± 11.29		
9~ 15	49.55 ± 9.06^a^			40.79 ± 11.53		
≥ 16	46.63 ± 8.87^ab^			41.60 ± 12.41		
Marital status		1.42	F = 2.29		0.15	F = 1.40
Unmarried	50.81 ± 9.27			41.50 ± 11.27		
Married, but separated	49.17 ± 9.48			41.67 ± 11.15		
Married, living together	49.62 ± 8.56			39.42 ± 11.91		
Education		0.91	F = 2.39		2.22	F = 0.87
High school diploma or below	50.04 ± 9.41			40.94 ± 11.35		
Some college	50.59 ± 9.30			41.89 ± 11.80		
Bachelor’s degree or above	51.71 ± 8.62			41.82 ± 10.44		
Residential Location		6.62**	Z = −0.91		0.09	F = 0.92
Rural area	50.46 ± 8.90			41.61 ± 11.27		
City	50.56 ± 9.83			40.92 ± 11.37		
Rank		1.61	F = 8.17**		1.66	F = 2.62*
Soldier	51.76 ± 9.17			41.85 ± 11.34		
Sergeant	49.50 ± 9.34^a^			40.84 ± 11.44		
Officer	51.90 ± 8.57^b^			42.49 ± 10.57^b^		
Household income(yuan/month)		1.59	F = 0.69		0.76	F = 2.92*
< 2000(~ 302 US dollar)	50.12 ± 9.64			40.45 ± 11.51		
2000 to 4000(~ 302 to 605 US dollar)	50.84 ± 8.74			41.82 ± 10.93		
> 4000(~ 605 US dollar)	50.63 ± 9.50			42.63 ± 11.64^a^		

Note: *p < 0.05, **p < 0.01; The ANOVA test was applied when variance homogeneity was satisfied (F-measure); non parametric rank test was applied when missing variance (Z-measure or Chi square value)

^a^Mean values were significantly different from those of the first group (*p* < 0.05)

^b^Mean values were significantly different from those of the second group (*p* < 0.05)

### Analysis of the health status relationship with deployment status, BMI and health-related behaviors

As shown in Table 5, the PCS and MCS scores showed significant association with all factors (*p* < 0.05, *p* < 0.01), with the exception of mean MCS score and deployment status (p>0.05). Moreover, participants with deployment experience displayed lower PCS score than those without. Similarly, the heavy smokers (≥20 cigarettes/day) or obese (BMI ≥ 28 kg/m^2^) participants had lower PCS and MCS scores, and were below the Chinese general population mean of 50. Also, the higher frequency of drinking or binge drinking correlated with lower PCS and MCS scores.

**Table 5:** Relationships of PCS and MCS scores with deployment status and health-related indicators of participants

Variable	PCS			MCS		
	Mean ± SD	Levene	F/*χ*^2^(Z)	Mean ± SD	Levene	F/*χ*^2^(Z)
Past deployment status		0.65	F = 5.74*		0.32	F = 0.16
Deployment experience	49.24 ± 8.90			41.65 ± 11.58		
No deployment experience	50.88 ± 9.31			41.32 ± 11.23		
BMI(kg/m^2^)		0.63	F = 3.86*		0.26	F = 2.76*
< 18.5	49.11 ± 10.12			45.09 ± 10.81		
18.5~ 23.9	50.99 ± 9.15			41.61 ± 11.08		
24.0~ 27.9	50.10 ± 9.14			40.74 ± 11.58^a^		
≥ 28.0	46.96 ± 9.99^bc^			39.40 ± 12.72^a^		
Smoking		2.16	F = 2.84*		1.60	F = 2.96*
Smoked<20cigarettes/day	50.11 ± 9.40			41.85 ± 10.91		
Smoked≥20cigarettes/day	46.86 ± 10.31			34.49 ± 10.61^a^		
Never smoked	51.33 ± 8.70^ab^			41.26 ± 11.57^b^		
Past smokers	49.37 ± 11.06			40.40 ± 12.40^b^		
Drinking		2.70*	*χ*^2^ = 8.56*		0.78	F = 8.15**
Everyday or almost everyday	48.15 ± 10.29			35.05 ± 11.24		
Frequent	49.46 ± 9.71			40.11 ± 11.30^a^		
Occasional	50.80 ± 9.16			42.49 ± 11.03^ab^		
Never	51.15 ± 8.67^a^			40.85 ± 11.53^a^		
Binge drinking		2.78*	*χ*^2^ = 16.66**		2.60	F = 5.56**
Everyday or almost everyday	43.68 ± 11.27			32.31 ± 10.79		
Frequent	48.79 ± 9.99^a^			39.29 ± 12.71^a^		
Occasional	50.02 ± 9.23^a^			41.67 ± 10.67^a^		
Never	51.40 ± 8.90^abc^			41.87 ± 11.31^ab^		

Note: *p < 0.05, **p < 0.01; The ANOVA test was applied when variance homogeneity was satisfied (F-measure); non parametric rank test was applied when missing variance (Z-measure or Chi square value)

^a^Mean values were significantly different from those of the first group (p < 0.05)

^b^Mean values were significantly different from those of the second group (p < 0.05)

^c^Mean values were significantly different from those of the third group (p < 0.05)

everyday or almost everyday = 6–7 times/week; Frequent = 1–5 times/week; Occasional = 3 times or below/month

### Multiple linear regression analysis of health status with sociodemographic characteristics, health-related behaviors and BMI

To further control the effect of known confounding factors such as age and education, we performed a multivariate linear regression analysis of the significant variables determined by univariate analysis, including the length of service, rank, household income, past deployment status, BMI, smoking, drinking and binge drinking, and the results have been summarized in Table 6. Among the different sociodemographic characteristics, the independent influencing factors that significantly correlated with PCS and MCS scores were length of service and household income, respectively. Participants with long service years were significantly more likely to have lower PCS score compared with soldiers who had a short service life. Similarly, the participants with high household income level were observed to have high MCS score.

**Table 6:** Multiple linear regression analysis of health status with sociodemographic characteristics, BMI and health behavior

health status	Variables	Beta	SE	Standardized Beta	Adjusted R Square	*t*	*p*	Durbin-Watson	F
PCS								1.603	7.95**
	Binge drinking	−2.454	1.069	−.074	0.011	−2.295	.022		
	Length of service	−1.384	.628	−.071	0.010	−2.204	.028		
	Regular drinking	−2.793	1.239	−.072	0.009	− 2.255	.024		
	BMI	−1.927	.879	−.070	0.009	−2.192	.029		
MCS								1.71	9.46**
	Binge drinking	−2.414	.658	−.112	0.003	3.671	.000		
	Household income	1.885	.671	.086	0.006	2.811	.005		
	BMI	−2.003	.799	−.077	0.007	−2.507	.012		

Note: **p < 0.01. Adjustments for race, age, education, marital status and residential location were made in the analysis

In terms of health-related behaviors and BMI, the independent influencing factors for PCS score were frequent drinking, binge drinking and BMI, while for MCS score, it was only binge drinking and BMI. Specially, the participants who were overweight or obese and accustomed to binge drinking had lower PCS and MCS scores, while participants who were accustomed to frequent drinking, only had lower PCS score.

### Logistic regression results predicting self-rated general health

All significant predictors from the univariate analysis were also entered into a final multivariable logistic regression model. As shown in Table 7, only the length of service, BMI, and binge drinking remained significant predictors for self-rated general health. Similar to the multiple linear regression model described above, individuals who were binge drinkers, had longer length of military service, and were overweight or obese, had higher risk of poor self-rated health than individuals who had no binge drinking habits, had a shorter service life, and normal BMI.

**Table 7:** Logistic regression results predicting self-rated general health

Predictor	Beta	SE	Wald	*p*	OR (95% CI)
Binge drinking	−0.251	0.154	3.943	0.047	0.737 (0.545 to 0.996)
Length of service (years)	−0.356	0.091	9.794	0.002	0.752 (0.629 to 0.899)
BMI	−0.285	0.118	4.507	0.034	0.778 (0.617 to 0.981)

Adjustments for race, age, education, marital status and residential location were made in the analysis

## Discussion

This cross-sectional study has investigated various determinants affecting the health status of Chinese navy personnel, using a specific population sample with an emphasis on health related behavioral factors. To our knowledge, this is the first study in China, to assess the navy personnel health status based on PCS and MCS scores, and their relationship with different health risk behaviors. This study would eventually have important implications for China’s public health system, especially for the health of Chinese military personnel.

In the present study, approximately 76% of the participants reported good health, indicating that most of navy soldiers and officers feel better about their health status. This information was consistent with study by Smith et al. [25], which showed that most respondents (69.8%) of US Navy and Marine Corps personnel reported their health as being very good or excellent. However, in the 8 domains of HRQOL, navy personnel had lower scores from 5 to 18 points than the general Chinese population aged 14~ 44 years, indicating that HRQOL in these domains was less favorable [26]. In comparison with US military samples of Millennium Cohort Study (where navy population accounted for 23.6%), our study participants showed 6 subscales that were lower from 4 to 13 points except for BP and VT subscale scores which tended to be better than that of US military samples [27].

Based on SF-36 V evaluation results, we observed that the PCS score was above the Chinese population mean of 50, indicating that the navy’s physical health status was better than general Chinese population. Moreover, the MCS score was about 8 points lower than general Chinese population. The reason may be the job environment and job characteristics that navy officers and soldiers confronted were under greater psychological pressure, and they could have higher probability suffering from mental disorder in comparison with people of similar age in Chinese population [28, 29]. Although the MCS score of the navy at the survey time was in the normal range considering their occupation, their mental health status may need extra attention to meet the requirements of modern military construction and high-tech war in the future.

Furthermore, in the present study we have identified relationship through multivariate model, of number of sociodemographic and military characteristics, including age, educational attainment, marital status, race, household income, residential location, rank, duration of military service, with health status. The results showed that the length of service and household income were independently associated with physical and mental health status, respectively. Importantly, we noticed that participants with long service time had lower PCS scores, thereby suggesting more physical health problems in these participants. The result was in line with the studies of Tian et al. [30] and Smith et al. [1]. Tian et al. found that longer military service correlated with lower score of physical functioning (p<0.001), while Smith et al. found that military members with longer lengths of service had less favorable physical health (p<0.05). Our finding is also independently confirmed with the observation that longer service times showed association with self-rated less than good health (poor). This could be attributed to the fact that navy personnel with long military service completed more tasks of combat training mission, and long period also exposure them to various health hazards and intense and stressful environments, thereby resulting in greater risk to their health.

Interestingly, the participants with higher household income displayed higher MCS score, representing more favorable mental health status than soldiers with less household income. Feng et al. [31] reported that family financial difficulties influenced the mental health status of navy officers and soldiers. The findings of our study are basically in agreement with previous studies. The reasons may be related to more psychological pressure on navy personnel with lower household income. If psychological pressure could not be alleviated, it would probably lead to poor mental health status. Thus, this observation in our study will have important implication for health professionals to identify those personnel, who are most likely in need of help, and eventually allocate the health care resources, accordingly.

Additionally, higher rates of health risk behaviors such as cigarette smoking and alcohol misuse among veterans have been reported in comparison to non-veterans in various studies [2, 3]. The smoking prevalence estimated by our study in navy personnel was lower than the civilian rate of 59.7% as reported by Xu et al. in 2010 [32], but it was significantly higher than prevalence of current smoking (17.9%) of navy personnel in Sri Lanka [33] and 2005 estimate of US Navy soldiers (32%) [34]. However, the navy personnel had significantly higher drinking rate than general population (39.6%) as reported by Ma et al. [35] in 2007. LeardMann et al. [36] documented that 32.6% of US Marine Corps recruits were high-risk and potentially problematic drinkers. Comparably, our study also showed that nearly 47% of the participants in our study have experienced binge drinking. The studies by Widome et al. [2] and Ryan et al. [37] have demonstrated that military personnel are more likely to have higher BMIs, and thus would be categorized into the overweight category. Similarly, our study observed that about 26% of the participants were overweight, which was higher than the overweight rate (22.8%) in Chinese population over the age of 18 years, as reported by a national survey in 2005 [38]. There are indications about overweight or obesity association with long-term drinking, smoking, and lack of exercise, and it has been observed that navy personnel while on duty have irrational dietary plans. For example, when the navy personnel are on duty at sea, they consume more red meat and processed meat, but fewer fruits and vegetables and also no engaged in regular exercise routine than during other time [39, 40].

Finally, our study also examined the relationship of health status with health-related behaviors, BMI and deployment status of navy members. Our analysis identified that binge drinking and BMI were independently associated with both physical and mental health status, whereas frequent drinking was independently associated only with physical health status outcomes. The participants with higher physical and mental health scores were observed to be involved in significantly less risky behaviors like binge drinking, regular drinking, and BMI, and reported significantly good self-rated health. It again confirmed that binge drinking and BMI were associated with self-rated fair/poor general health. Overall it can be deduced that increased awareness about the health hazards of alcohol misuse should be emphasized and some alcohol reduction strategies for health promotion should be introduced. Moreover, effective measures on modifying BMI in military need to be carried out and implemented. Relevant departments need to invest the necessary funds to improve the living facilities and environmental conditions of the troops, especially provide adequate fresh vegetables and fruits to soldiers on duty or training at sea.

Although deployment status and smoking were significant predictors in the preliminary variance analysis model, they did not remain significant in the final analysis. This observation was contrary to the results of studies by Ryan et al. [37] and Robyn et al. [15] and Jahnke et al. [6]. Ryan and Robyn suggested that the psychological and physical effects of deployment may have a greater impact on health. At least some military activities might be resulting in mental and physical health deterioration. In a population-based study conducted on U.S. Marines, Robyn et al. [15] found that significant number of individuals in the war-deployed cohort were likely to have a posttraumatic stress disorder (PTSD) diagnosis than individuals in the non-war-deployed cohort (p<0.01). The HRQOL reported by these veterans has also been shown to be significantly less favorable [41]. However, in the present study, deployment status had no significant effect on either the health scores of PCS, MCS or self-rated general health. This difference could be attributed to the fact that the participants in their study were mainly involved in war-related military operations, such as Gulf War, Vietnam War and Operation Iraqi Freedom, while Chinese navy personnel has spent relatively little time waging conventional battles and has only engaged on numerous non-war military actions, for instance, peacekeeping operations, earthquake relief actions, security operations.

The study by Jahnke et al. [6] analyzed the impact of tobacco use on military health and readiness. Interestingly, we observed no significant association of smoking with PCS and MCS scores in the final model, and this observation was consistent with the finding by Darviri et al. [42], who also showed that smoking did not exhibit significant association with self-rated health. Again, this could partly be due to the fact that (1) navy soldiers are young, and the disease caused by smoking takes a long time to show; and (2) previous study have shown that smoking is considered to be an accepted stress relief method. However, further studies would be required to help confirm the specific reasons.

Our study has certain limitations. Firstly, it is a cross-sectional study and thus we cannot make definitive conclusion about the observed associations. Secondly, weight was recorded in a self-report form and was likely to be underestimated. This would result in underreporting of BMI. Thirdly, there were 117 participants who did not provide complete information. However, their characteristics were not significantly different from those included in the analysis, and non-responders are not in high number. Thus, we expect that exclusion of these non-responders did not influence the statistical analyses. Moreover, sample size was not determined, since it could meet the requirements for ANOVA and regression analysis.

## Conclusion

In conclusion, our study provided the preliminary information on the health status (based on physical and mental scores) and various factors affecting it in Chinese navy personnel. Our study demonstrated that some sociodemographic and health-related behavioral factors such as, length of service, frequently drinking, binge drinking and BMI, influence the health status. This initial baseline information would be helpful for designing the health promotion strategy for military personnel in China and assisting health policy makers in preparing for appropriate health, nutrition, occupational environment, and social support guideline for the Chinese navy personnel. However, further research would be required by including large data points to specifically focus on the different health risk factors, and evaluating the dynamic changes in the health status. These interventions will definitely help to improve the overall health of the navy personnel and subsequently improving their combat capability.

## Abbreviations

MCS: Mental component summary
PCS: Physical component summary
SF-36: Short form 36
